# Hydrothermal-Assisted Synthesis of Copper Nanoparticles-Decorated Titania Nanofibers for Methylene Blue Photodegradation and Catalyst for Sodium Borohydride Dehydrogenation

**DOI:** 10.3390/polym14235180

**Published:** 2022-11-28

**Authors:** Ahmed Abutaleb

**Affiliations:** Department of Chemical Engineering, College of Engineering, Jazan University, Jazan 11451, Saudi Arabia; azabutaleb@jazanu.edu.sa

**Keywords:** copper nanoparticles–TiO_2_ nanofibers, methylene blue, photocatalyst, sodium borohydride

## Abstract

Simple and inexpensive electrospinning and hydrothermal techniques were used to synthesize titania nanofibers (TiO_2_ NFs) (composite NFs) decorated with copper nanoparticle (Cu NPs). The fabricated composite NFs have been tested as a photocatalytic material to degrade methylene blue (MB) as a model dye under visible light. The introduced composite NFs have shown good photocatalytic activity compared with pristine TiO_2_ NFs; 100% and 50% of dye were degraded in 120 min for composite NFs and pristine TiO_2_ NFs, respectively. Furthermore, composite NFs demonstrated good stability for four cycles. In addition, the fabricated Cu-TiO_2_ NFs have shown good photocatalytic activity for the production of H_2_ from sodium borohydride.

## 1. Introduction

The increase in water pollution, due to toxic organic pollutants, discharged from various industries, is harmful to human health and aquatic life systems, and causes aesthetic pollution problems [1,2,3]. In particular, the textile industry releases a large volume of water after dying and washing cotton fibers to remove any unfixed residual dyes [4,5,6]. The draining of colored wastewater into water resources such as rivers inhibits light passing and decreases dissolved oxygen in the water. These impacts on water negatively affect aquatic life [7,8]. Different traditional techniques have been used to remove the color of dyes (e.g., adsorption, flocculation, and coagulation) [6,7,8]. These processes cannot completely remove the dyes, but can only separate the dyes as sludge. This creates a secondary pollution problem [9,10]. Thus, a further treatment step is needed to remove the sludge from the purified water. The photocatalytic reaction approach, photodegradation, is widely recognized as a reliable strategy for de-colorizing wastewater without causing any further environmental harm [10,11,12]. Photodegradation is potent in environmental remediation since it converts toxic organic pollutants in water and air into non-toxic products. It is necessary to run the photodegradation process in the presence of a catalyst for heterogeneous metal oxides and a light source (visible or UV light) [13,14,15]. It is well known that titanium dioxide (TiO_2_) photocatalyst is the most applied catalyst in organic pollutants decomposition [16]. TiO_2_ possesses many advantages, such as its resistance to biological and chemical attacks, high oxidizing potential, inexpensiveness, environmental conscientiousness, and long-term resistance against chemical and photo corrosion [16,17]. Since TiO_2_ has a very large band gap (3.3 eV), photogenerated electrons and holes quickly recombine during the photodegradation process, causing the material to degrade [18,19]. As a result, its photodegradation processes have a poor quantum yield, reducing its photocatalytic efficacy [10,20]. To address this challenge and boost the commercialization of the photocatalytic method for degrading hazardous organic pollutants, several co-catalyst metal NPs are combined with TiO_2_ [21,22,23]. One way to improve the separation of holes and photogenerated electrons is to couple metal NPs with TiO_2_, which leads to band gap tuning [10]. Electrons are easily trapped by metal NPs due to their lower Fermi level compared to TiO_2_, whereas the holes are set at the valance band of TiO_2_, which produces highly intensive free radicals that degrade the organic pollutants in the solutions [10,24]. Furthermore, the fabrication protocol has a direct effect on the photocatalytic process. In this study, metallic copper NPs are incorporated in TiO_2_ nanofibers (NFs) as an efficient photocatalyst for the degradation of methylene blue. The photocatalyst is prepared using electrospinning and hydrothermal processes. The introduced photocatalyst showed complete degradation of MB as a model dye under the sunlight within 60 min. In addition, the prepared photocatalyst showed good catalytic activity towards hydrogen release from sodium borohydride.

## 2. Materials and Methods

### 2.1. Materials

Titanium isopropoxide (TIPP, 97%), poly (vinyl pyrrolidone) (PVP, MW = 13,000.00 g·mol^−1^), acetic acid (AA), and ethanol were purchased from Sigma Aldrich, St. Louis, MO, USA, and used as purchased, and used as purchased [14,17]. Copper (II) nitrate trihydrate solution (MW) was purchased from Junsei Chemical Co., Ltd., Chuo-ku, Tokyo.

### 2.2. TiO_2_ Nanofibers Preparation

TiO_2_ NFs were prepared using electrospinning followed by calcination at a high temperature. The catalytic fabrication process includes the following steps: (1) preparation of electrospinning homogenous solution, (2) electrospinning of the prepared solution, and (3) calcination of the polymeric electrospun NFs.

To prepare the electrospinning solution, first, a 15 wt% PVP polymer solution was prepared by dissolving 1.5 g PVP powder in a solvent mixture of AA and ethanol (50:50 wt%). The PVP solution was perfectly mixed at room temperature using a magnetic stirrer until a homogenous polymer solution was obtained. Then, 1 g of TIPP was added to the previous homogenous PVP solution. Continuous mixing of the (TIIP-PVP) electrospinning solution at room temperature was performed until a homogenous yellow transparent (TIIP-PVP) sol–gel was achieved.

Second, the electrospinning solution was converted into polymeric NFs using lab scale electrospinning machine. This process was started by injecting the prepared yellow sol–gel into a plastic syringe. The stainless-steel tip of the syringe was connected to the positive pole of a power supply, whereas the negative pole was connected to a rotating cylinder covered with a poly(ethylene) sheet. The electrospinning operating condition was fixed at a 15 cm working distance between the rotating drum and the needle tip, room temperature, 20 kV, and 0.8 mL h^−1^ flow rate. The obtained TIIP-PVP NFs sheet was vacuum-dried at 60 °C for a full day to ensure the evaporation of the residual solvent.

Finally, the calcination process was performed for 2 h at 700 °C to remove the PVP polymer and convert TIIP into TiO_2_ NFs.

### 2.3. Cu-Doped TiO_2_ Nanofibers Preparation

To prepare Cu-doped TiO_2_ NFs, 1 g of prepared TiO_2_ NFs was added to 0.025 M copper (II) nitrate trihydrate solution. The solution was stirred for 1 h at room temperature to disperse the Cu precursor particles on the surface of the TiO_2_ NFs. Finally, a reduction process was performed to convert the Cu precursor to Cu nanoparticles (NPs). Hydrazine hydrate (750 µL) and formic acid (750 µL) were drowsily added to reduce the previous solution [25,26]. The solution was stirred for 30 min at room temperature. The solution was finally transferred to an autoclave (Teflon-lined stainless steel) that was heated at 170 °C for 6 h in a muffle furnace. Finally, to clean the formed catalyst from impurities, it was filtrated and washed with ethanol and water, then dried at 80 °C for 24 h.

### 2.4. Characterization

The morphology of the fabricated electrospun catalyst was tested using a field emission scanning electron microscope (FESEM, Hitachi S-7400, Tokyo, Japan). A transmission electron microscope (TEM, JEOL Ltd., Tokyo, Japan) operating at 200 kV with EDX. UV-visible spectroscopy was also used to examine the concentration of dyes during the photodegradation process (HP 8453, Berlin, Germany).

### 2.5. Photocatalytic Activity Test

The photodegradation study of MB was executed in a batch reactor (glass bottle with 100 mL capacity). In total, 25 mg from the prepared photocatalysts was added to 50 mL of the 10 ppm MB solution and exposed for sunlight irradiation. The temperature of the solar irradiation was 29 ± 1 °C, with continuous stirring. In total, 2 mL from the solution was taken out at specific intervals and centrifuged to separate the residual NF catalyst. The absorbance of the separated MB solution was measured using a UV-visible spectrophotometer at λmax = 664 nm. A calibration curve was constructed between different concentrations and absorbance to measure unknown concentrations at defined absorbance.

### 2.6. Catalytic Hydrolysis of SBH

Using a lab-scale reactor, as reported in previous studies [27,28,29,30,31,32], hydrogen was released from SBH. An alkaline solution of 100 mM SBH was introduced to the reactor, along with a determined amount of Cu-TiO_2_ catalyst (37.83 mg, 1 mmol in 10 mL solution). The reactor was hooked up to a graduated cylinder containing water, and a magnet was used to agitate the solution at 900 rpm. The quantity of H_2_ released from SBH was calculated by monitoring the fall in water level in a graduated cylinder.

## 3. Results and Discussion

**Figure 1:**
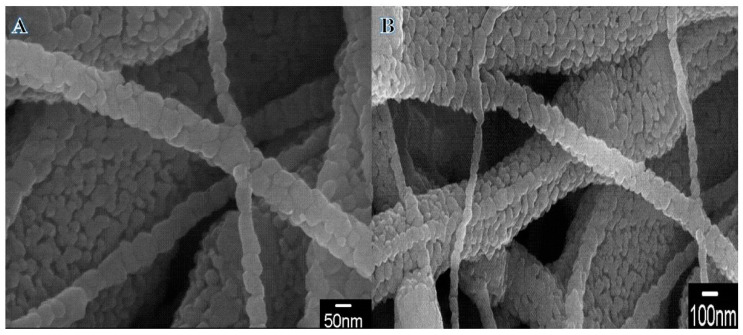
Low and high magnification FE-SEM images of the produced powder after hydrothermal process.

**Figure 2:**
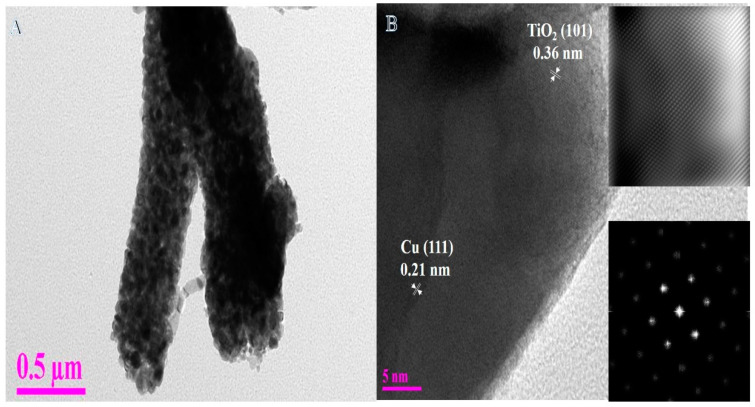
(**A**) Normal TEM image and (**B**) HR-TEM image of produced powder after the hydrothermal process (Inset in b shows the SAED image).

**Figure 3:**
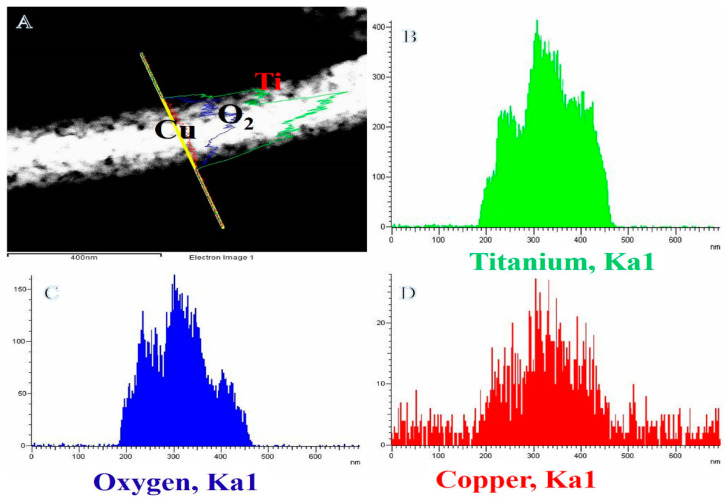
(**A**) STEM image along with the line EDX analysis of the produced powder after the hydrothermal process; (**B**–**D**) indicate line analysis EDX for the line shown in (**A**).

Based on its ease of use, cheap cost, high yield, and high efficiency, electrospinning stands out as the best method for producing NFs [25,33,34,35,36,37,38,39,40,41,42,43,44,45]. As known, size and morphology have direct effects on the chemical and physical properties of materials. The produced NFs have a diameter in the ranges of 50 to 1000 nm. The nanofibrous structure has a high axial ratio which could improve the catalytic performance in different reactions. After undergoing hydrothermal treatment, the NFs are shown at low and high magnification in FSEM images (Figure 1A,B, respectively). As a result of the process’s vigorous chemical reactions, a good nanofibrous structure is preserved. In addition, the high temperature, pressure, and reduction reaction that occur throughout the procedure allow nanopores to be seen extremely clearly in the resulting images. More morphological details of the prepared NFs were investigated by TEM and HRTEM images (Figure 2A,B). A normal TEM image (Figure 2A) shows the formation of hetero-structure NFs. According to the deposition process, the Cu NPs covered the surface of TiO_2_ NFs. The lattice fringes of NFs were determined to be 0.36 and 0.21 nm (Figure 2B), whose obtained values correspond to the TiO_2_ (101) and metallic Cu (111) planes, respectively [5,6]. Furthermore, few Cu species might be interstitial to the TiO_2_ matrix due to the thermally enhanced diffusion process. The respective HRTEM images (Figure 2B) indicated the formation of highly crystalline material. The image shows the formation of an interfacial region between Cu NPs, and the matrix is clear in the image. The inset in Figure 2B represents the SAED pattern which confirmed the formation of highly crystalline composite material without any defects. Figure 3 displays the TEM-EDX image for one selected NF; it confirmed the formation of hetero-structure NF with the rough and nano-porous surface.

Figure 3B–D indicate the EDX analysis of the drawn line in Figure 2A. The EDX analysis shows the formation of Cu, Ti, and O elements only. The copper NPs are homogeneity distributed along the Titania NF and its surface. In other words, copper NPs are grown at Titania matrix-like core–shell structure due to the deposition process; the interfacial region confirms this in the HRTEM image (Figure 2B).

**Figure 4:**
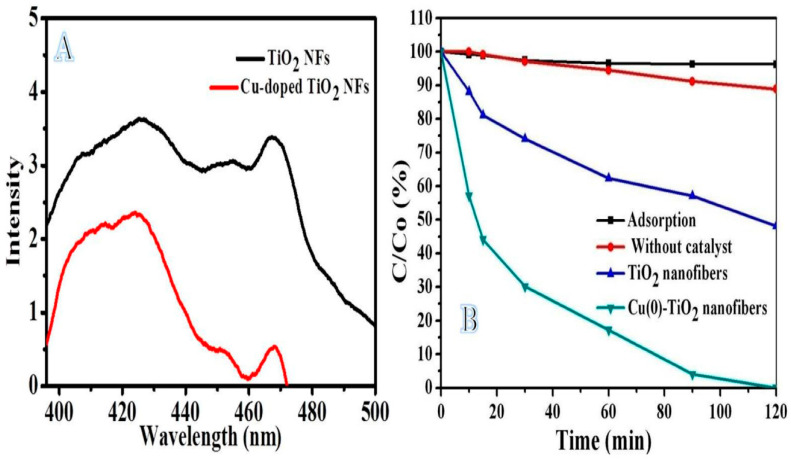
Photoluminescence spectrum of composite NFs and TiO_2_ NFs (**A**) and photodegradation profiles of the MB under sunlight irradiation (**B**).

The PL Emission spectra were used to investigate the semiconductor lifetime and charge separation in which PL indicates the electron/hole recombination rate in the semiconductor materials. The comparison of the PL spectrum (applied ʎ = 320 nm) of Titania NFs and copper-doped Titania NFs shows a similar spectrum at emission peaks 422 and 468 nm (Figure 4A). As seen in figure, copper-doped Titania NFs showed a lower intensity peak compared to Titania NFs which demonstrates a lower electrons/holes recombination rate and low defects in the copper-doped Titania NFs [46]. This might be attributed to the excited electrons from the valance band of Titania NFs to their conduction band and finally transfer to co-catalytic Cu NPs, which inhibit the electrons/holes recombination. This is desirable in exploiting the materials in the photocatalytic reaction.

Figure 4B shows the photodegradation study of MB used to prepare NFs to evaluate their activity. The copper-doped Titania NFs have shown a good photocatalytic performance compared with Titania NFs as 100% and 52% of dye have been removed at 120 min, respectively, under sunlight irradiation. It is evident from the PL result that copper-doped Titania NFs can be effectively used under visible light; however, pure Titania NFs can only be used as a photocatalyst in the ultraviolet spectrum [13]. For comparison, the adsorption effect of prepared NFs and photocatalytic activity without photocatalyst were studied. They have not shown any observable effect in dye degradation

**Figure 5:**
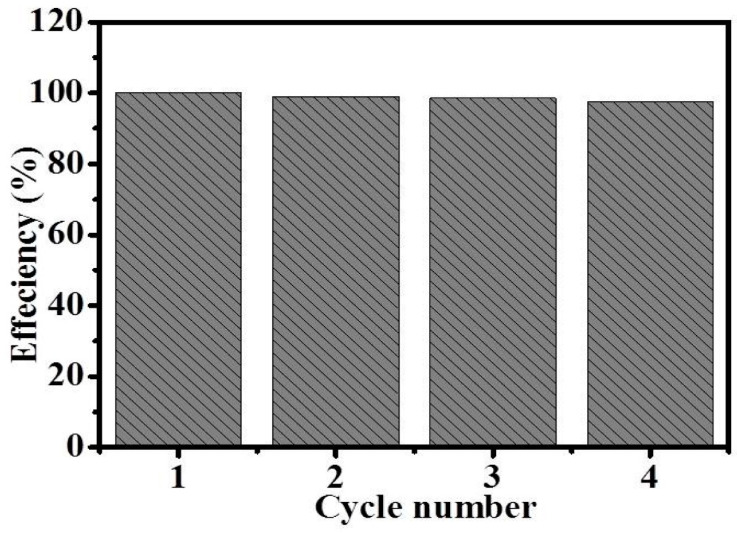
Reusability test of composite NFs.

To study the long performance of fabricated copper-doped Titania NFs, photocatalytic NFs were used for four cycles (Figure 5). As shown in Figure 4 below, good a photocatalytic response with little change in the catalytic performance was observed, which demonstrated that the introduced photocatalytic NFs are robust in the catalytic reaction.

### Hydrolytic Dehydrogenation of NaBH_4_ Using Cu-TiO_2_ Catalyst

**Figure 6:**
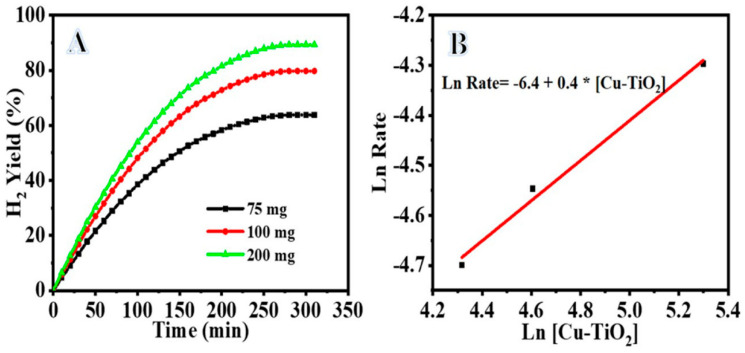
(**A**) Effect of Cu−TiO_2_ amount on hydrogen generation from aqueous NaBH_4_; (**B**) plot of logarithmic hydrogen generation rate vs. Cu−TiO_2_ amount.

**Figure 7:**
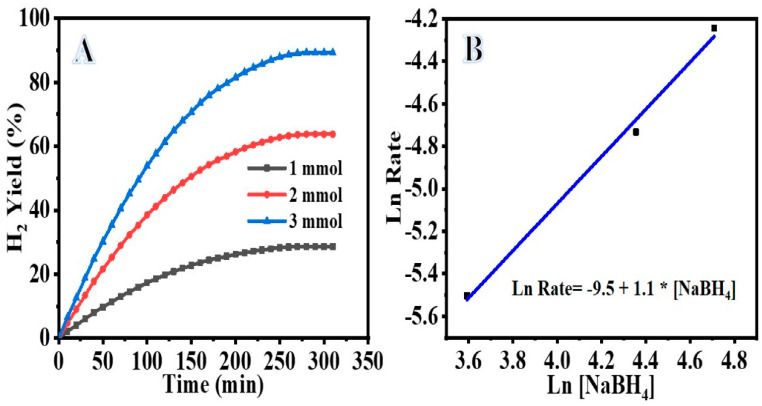
(**A**) Effect of NaBH_4_ concentration on the catalytic performance; (**B**) plot of logarithmic of hydrogen generation rate vs. [NaBH_4_].

**Figure 8:**
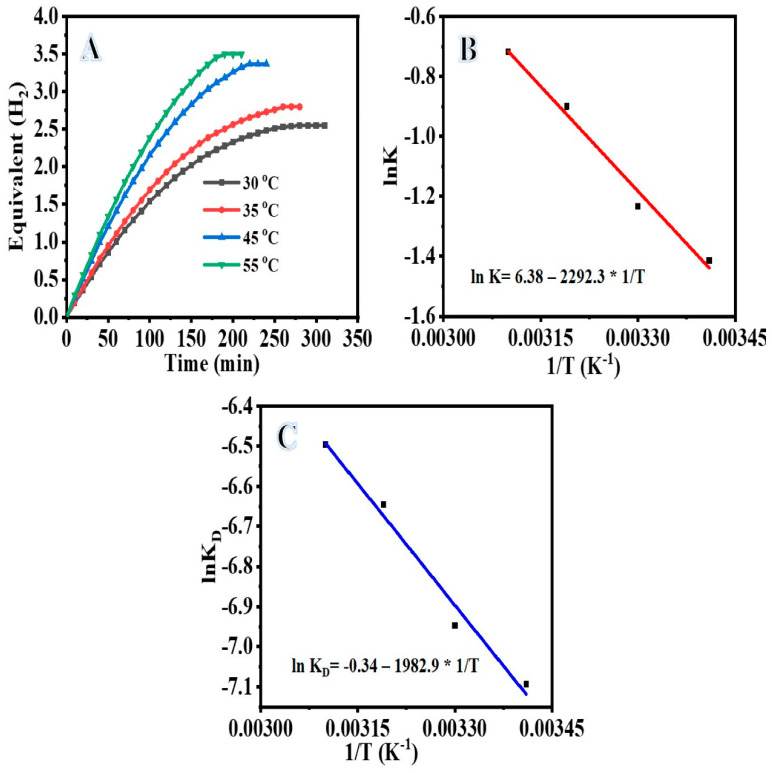
(**A**) Effect of temperature on hydrogen released from aqueous NaBH_4_ in the presence of Cu-TiO_2_; (**B**) plot of ln K vs. 1/T; (**C**) plot of ln (K/T) vs. 1/T.

Metal hydrides such as ammonia borane (NH_3_BH_3_), hydrazine hydrate (N_2_H_4_.H_2_O), and sodium borohydride (NaBH_4_, SB) are very promising H_2_ storage materials due to their gravimetric density and high capacity for H_2_ storage [47,48]. SB is considered one of the best metal hydride H_2_ storage materials with 10.8% H_2_ capacity by weight. The hydrolysis of SB with water in the presence of an effective catalyst resulted in an exothermic reaction that produces H_2_. The reaction can occur at a low temperature to produce pure and controllable H_2_. SB hydrolysis reaction is irreversible and generates four moles of H_2_. Recently, Cu@TiO_2_ nanostructures have demonstrated excellent catalytic performance towards H_2_ generation from hydrogen storage materials [49,50]. Here, first, a controlled experiment was performed to demonstrate that at 30 ± 1 °C and 55 ± 1 °C, bare TiO_2_ had no catalytic activity in the hydrolysis process. Figure 6A depicts the percentage of H_2_ gas produced from the NaBH_4_ hydrolysis as a function of the reaction time in the presence of different amounts of the fabricated catalyst (Cu-TiO_2_) (75 mg, 100 mg, and 200 mg) and 1 mmol NaBH_4_ at 30 ± 1 °C. When Cu-TiO_2_ was added to the reactor, the NaBH_4_ hydrolysis began immediately. Increases in catalyst amount resulted in sustained hydrogen release for longer times. Accordingly, the rate at which hydrogen is produced is proportional to the amount of the catalyst. NaBH_4_ hydrolysis follows half-order kinetics with regard to the amount of Cu-TiO_2_ as shown by the straight line with a slope of 0.4 in Figure 6B. Figure 6B plots the rate of H_2_ production against the logarithmic values of Cu-TiO_2_ amount. Figure 7A shows the NaBH_4_ concentration (1 mmol, 2 mmol, and 3 mmol) and its influence on hydrogen production rate. Both the catalyst dose (75 mg) and the temperature (30 ± 1 °C) were kept constant. As shown in Figure 7A, as the concentration of NaBH_4_ increases, so does the rate at which hydrogen is produced. NaBH_4_ hydrolysis is first order in its dependence on [NaBH_4_], as seen by the straight line with a slope of 1.1 in a plot of hydrogen production rate vs. logarithmic values of [NaBH_4_] *(*Figure 7B). The activation energy (Ea) of Cu-TiO_2_ in the NaBH_4_ hydrolysis process was measured by catalyzing the reaction using Cu-TiO_2_ = 75 mg and 1 mmole NaBH_4_ at different temperatures (Figure 8A). Increasing the reaction temperature results in a relatively larger hydrogen generation. By plotting the logarithmic *k* and *K_D_* values as a function of the inverse temperature (1/T), where *k* is the rate constant for hydrogen generation, the Arrhenius and Eyring plots (shown in Figure 8B,C) can be constructed using Equations (1) and (2), respectively.

(1) $$k=Ae^{\frac{-E_{a}}{RT}}$$

(2) $$\ln K_{D}=\left(\frac{\Delta S{}^{\circ}}{R}\right)-\left(\frac{\Delta H{}^{\circ}}{RT}\right)$$

From the Arrhenius and Eyring plots, we determine that the reaction thermodynamic parameters (*Ea*, *S*, and *H*) are 19.03, 0.02894, and 16.48 kJ/mol.

## 4. Conclusions

Cu-NP-decorated TiO_2_ NFs were successfully prepared using a simple electrospinning technique followed by a hydrothermal process. The characterization technique confirmed the deposition of CuNPs on the surface of TiO_2_ NFs. PL data showed the lower recombination of electrons and holes compared to TiO_2_ NFs. Accordingly, composite NFs have been shown to have good photocatalytic performance compared with TiO_2_ NFs. The introduced composite NFs have shown good photocatalytic activity compared with pristine TiO_2_ NFs; 100% and 50% of dye are degraded in 120 min for Composite NFs and pristine TiO_2_ NFs, respectively. In addition, the fabricated Cu-TiO_2_ NFs have shown good photocatalytic activity for the production of H_2_ from sodium borohydride. The H_2_ generation yield has been increased from 63% to 90% with an increase in the catalyst amount from 75 mg to 200 mg in the presence of 1 mmol sodium borohydride and 30 °C. This indicated that the reaction is catalyst-dependent. The kinetics study showed that the reaction followed the pseudo first-order reaction with respect to the concentration of sodium borohydride. The hydrogen generation has been increased with the increase in reaction temperature. A low activation energy (19.03 kJ mol^−1^) is obtained.
